# Navigating healthcare access and utilization in rural and Northern Ontario: Immigrants’ experiences amid regionalization and dispersal policies

**DOI:** 10.1371/journal.pone.0362085

**Published:** 2026-11-11

**Authors:** Aimable Nkurunziza, Morris D. C. Komakech, Abukari Kwame, Barbara Twum-Antwi, Mohammed Iddrisu, Namentha Srikanthan, Osman Muna, Geoffrey Maina

**Affiliations:** 1 Arthur Labatt Family School of Nursing, Western University, London, ON, Canada; 2 School of Nursing, Nipissing University, North Bay, ON, Canada; 3 College of Nursing Prince Albert Campus, University of Saskatchewan, Prince Albert, Saskatchewan, Canada; 4 College of Nursing Saskatoon Campus, University of Saskatchewan, Saskatoon, Saskatchewan, Canada; 5 School of Psychology, University of Ottawa, Ottawa, ON, Canada; Universitas Prima Indonesia, INDONESIA

## Abstract

**Background:**

Immigration to Canada has historically concentrated in major metropolitan areas, with over half of newcomers settling in Ontario’s urban centres in 2019. However, recent government initiatives seek to address labour shortages and stimulate regional development by encouraging immigrant settlement in five rural Northern communities. As policies increasingly direct newcomers to these regions, it becomes essential to understand their unique settlement experiences and ensure that local services effectively meet their needs, especially healthcare needs. Yet, the limited research focusing on immigration in rural and northern contexts constrains the development of evidence‑based programs and supports.

**Objective:**

To explore the experiences of immigrants in accessing and utilizing healthcare services in rural and northern Ontario in the context of post-government regionalization and dispersal policies.

**Methods:**

This qualitative descriptive study was conducted in North Bay, Ontario. Purposive sampling was employed to recruit 20 immigrants who had settled in North Bay through the Rural Community Immigration Pilot, along with four key informants who work with immigrant populations, to participate in virtual interviews. Data were analyzed thematically. Ethical approval was obtained from the Nipissing University Research Ethics Board (File No. 103946) on April 17, 2025.

**Findings:**

Findings indicate that healthcare access for immigrants is not a single encounter, but an ongoing, complex care journey shaped by barriers before, during, and after contact with services. Four interrelated themes characterized this journey: Getting In, which reflected challenges initiating care due to information gaps, limited primary care attachment, transportation and distance barriers, language readiness, and fear of discrimination; Getting Through, which captured participants’ struggles to obtain timely, understandable, respectful, and culturally safe care amid language barriers, long waits, fragmented referrals, and rushed encounters; Getting On, which described the post-visit consequences of unclear follow-up, diminished trust, and constrained self-management; and Getting the System Better, which highlighted participants’ recommendations for improving immigrant healthcare access through earlier orientation, peer navigation, transportation and financial supports, targeted immigrants services, reliable language access, multilingual resources, cultural competence training, and stronger follow-up systems.

**Conclusion:**

This study underscores the need to move beyond narrow definitions of healthcare access and toward a more holistic, immigrant-responsive model of care. Improving access requires proactive, coordinated, and culturally safe health system supports that build trust, strengthen continuity, and ensure that immigrants are not only able to reach services but are also meaningfully supported throughout their care journey.

## Introduction

Canada has a rich history of immigration, with millions of individuals from around the world choosing it as their new home. In 2021, over 8.3 million people, representing nearly a quarter (23.0%) of the population, were either current or former landed immigrants or permanent residents [1]. This marked the highest proportion since Confederation, surpassing the previous record of 22.3% set in 1921, and was the highest among G7 countries [1]. In 2022, Ontario welcomed a record-breaking 227,424 immigrants, solidifying its position as Canada’s top destination for newcomers. While over half of the immigrants settled in Ontario’s metropolitan areas in 2019 [2], recent policies aim to address labour shortages and foster local economies by incentivizing newcomers to settle in five rural communities in Northern Ontario [3]. These incentives offer skilled foreign workers a pathway to permanent residence and access to employment opportunities in participating communities.

North Bay is one of 11 rural communities selected for immigrant resettlement under the Government’s regionalization and dispersal policies [3]. According to the 2021 Census, 2,610 individuals, representing 5.1% of the population in North Bay, were foreign‑born immigrants [4]. Given resettlement policies are increasingly directing newcomers to Northern communities, and recognizing immigrants often face distinct settlement challenges in these settings, it is critically important to explore their experiences to ensure that services are responsive to their needs.

A scoping review of Canadian research on settlement to rural and smaller communities highlights several structural challenges that shape newcomer settlement. Rural areas tend to have less cultural and ethnic diversity, few local services, and geographically distant compared to major urban centre, making integration more complex for newcomers seeking familiar cultural networks and create barriers to accessing essential supports. Furthermore, their low population density often translates into reduced government funding, limiting the resources available to meet the needs of immigrant populations [5].

While immigrants in North Bay are satisfied with access to sociocultural, telecommunications, and education services, there was notably lower satisfaction with public transportation and healthcare [6]. The lower satisfaction with healthcare could negatively impact the mental well-being of immigrants, as access to reliable services is crucial for reducing stress, fostering a sense of security, and supporting overall integration into the community. In addition, immigrants and newcomers in Canada are at heightened risk for developing negative mental health outcomes due to social determinants of health and the healthy immigrant effect [7].

A dearth of literature focusing on the experiences and benefits of immigration in rural, remote, and northern areas impacts the ability to develop empirically informed policies and support programs for newcomers [8]. Thus, this paper explores the experiences of immigrants in accessing and utilizing healthcare services in rural and northern Ontario in the context of post-government regionalization and dispersal policies. A study of this nature is critical to gaining insights on the settlement and integration of newcomers, especially beneficiaries of the Rural Community Immigration Pilot schemes. Thus, the findings from this project will inform the specific health services needs of immigrants in rural and northern Ontario and guide the development of targeted interventions tailored to immigrants’ priorities.

## Methods

### Design

This study used a qualitative descriptive design. We adopted a descriptive exploratory approach to generate rich narrative data from a small sample and to develop in-depth insight into immigrants’ experiences navigating healthcare services in rural and northern Ontario [9]. The study was informed by a constructivist paradigm, recognizing that experiences and meanings are co-constructed through participants’ accounts and the research process [10]. We reported the study in accordance with the Consolidated Criteria for Reporting Qualitative Research (COREQ) checklist.

### Setting

The study was conducted in North Bay, Ontario. North Bay is one of the northern Ontario communities selected for immigrant resettlement under the Government’s regionalization and dispersal policies. North Bay is strategically situated within a three-and-a-half-hour drive of major Ontario hubs, including Toronto and Ottawa. According to the 2021 Census, the city has a population of approximately 52,662 and serves a broader regional market of about 112,000 people, encompassing the districts of Nipissing, Parry Sound, Timmins, Temiskaming Shores, and Cobalt [11]. Currently, immigrants represent approximately 5% of the total population [4].

North Bay’s healthcare system includes primary care provided by family physicians, walk-in clinics, public health services through the North Bay Parry Sound District Health Unit, and acute and mental healthcare services through the North Bay Regional Health Centre. Medically necessary physician and hospital services are publicly funded for eligible residents through Ontario’s healthcare system. However, newcomers may encounter challenges related to health insurance eligibility, securing a family physician, and accessing services not fully covered by public insurance, such as certain medications and allied health services [12].

### Participants’ recruitment

Two research assistants (RAs) were hired and trained to support participant recruitment and data collection. Purposive sampling was used to recruit 20 immigrants and four key informants. Following ethical approval (see below), immigrant participants were recruited using a study poster shared through immigrant associations in North Bay, social media platforms, immigrant-serving organizations, immigrants’ association, churches, markets, and the North Bay Parry Sound District Health Unit. Immigrants were eligible if they: (1) had immigrated through northern community immigration programs, (2) were 18 years or older, (3) had lived in the North for at least one year, (4) had accessed and used healthcare services in North Bay, and (5) could speak in English or French. Participants were excluded if, despite meeting the inclusion criteria, they were unable to provide informed consent, were experiencing acute distress or illness that would make participation unsafe, had previously participated in the study, participation might expose them to undue personal, legal, immigration-related, or emotional risk. Key informants were individuals who worked with immigrants within northern healthcare services. With support from the study advisory committee, key informants were identified through project meetings, Google searches, the North Bay Parry Sound District Health Unit, and local healthcare settings, and were recruited via email.

### Data collection procedures

Data were collected from May 1 to September 30, 2025. After eligibility screening, RAs emailed participants a letter of information and consent form outlining the study details. Participants were given two weeks to review the information, ask questions, and decide whether to participate. After providing written consent, participants returned the signed consent forms to the RAs and an individual interview was scheduled via Microsoft Teams or Zoom, or in person depending on participant preference; however, all interviews were conducted online. The interview guides (“S1 Appendix A” and “S2 Appendix B”) were developed by the study team, drawing on team members lived experiences as immigrants accessing healthcare in Canada, as well as extensive qualitative research experience with immigrant populations. With participants’ permission, interviews were audio-recorded and transcribed. Field notes were taken during and after each interview. Interviews lasted an average of 48 minutes. At the end of the interview, immigrant participants received a $50 gift card, and key informants received a $25 gift card as a token of appreciation for their time and internet costs. Once transcripts were retrieved, all audio recordings were permanently deleted from the RAs’ computers, including from deleted-items folders.

### Data analysis

Data were organized in Dedoose and analyzed using a thematic analysis framework [13–15]. Field notes were integrated into the transcripts to support interpretation. Two members of the research team coded the data and then met to compare codes and resolve discrepancies through discussion. Codes were subsequently grouped into categories and then refined into themes. The research team reviewed and agreed upon the major themes prior to reporting. Preliminary findings were presented to 21 study participants during a 90-minute online member-checking session to ensure the findings reflected their experiences. To enhance rigor, we attended to credibility, transferability, dependability, and confirmability [16].

Credibility was achieved through regular reflexive discussions, debriefing sessions with the RAs, and member checking to ensure that emerging interpretations remained grounded in participants’ accounts. Meetings with the advisory board provided ongoing guidance and helped validate the relevance and accuracy of the findings. Transferability was supported by capturing rich contextual detail through field notes and memos, enabling readers to assess how the findings may relate to other Northern or rural contexts. Dependability was enhanced through systematic double coding and routine documentation of methodological decisions, ensuring a transparent and consistent analytic process. Finally, confirmability was achieved through triangulation across diverse data sources, including interviews with immigrants and key informants, alongside the use of reflexive journaling and careful retention of analytic records, reducing researcher bias and grounding interpretations firmly in the data [16].

### Ethical considerations

Ethical approval was obtained from the Nipissing University Research Ethics Board (File No. 103946) on April 17, 2025. Written informed consent was obtained from all participants after they reviewed and understood the information provided in the study letter. Participants were informed that participation was voluntary and that declining to participate or withdrawing at any time would involve no adverse consequences.

## Findings

### Socio-demographic characteristics of the study participants (n=20)

A total of 20 immigrant participants (n = 20; 14 men and 6 women), ranging in age from 25 to 40 years with diverse socio-demographic backgrounds took part in this study and had lived in Canada for periods ranging from a few months to 20 years. The sample included individuals identifying as Black, Black American, White, Mixed-race, African, and Arabic backgrounds. Educational attainment varied from high school completion to master’s-level degrees, with English being the most common home language. Family structures varied, including individuals living alone, with spouses and children, or with extended relatives. Employment ranged from unemployed to full-time, with part-time employment being most common (Table 1).

**Table 1:** Socio-demographic characteristics of immigrant participants (n = 20).

ParticipantID	Agegroup	Gender	Relationshipstatus	Living arrangement	Education	Home-languagecategory	Racial/culturalgroup	Employment	Time inCanada
**P01**	25-29	Female	Not partnered	Lives alone	Bachelor’s	Other/non-English	Black	Part-time	2-5 years
**P02**	25-29	Female	Not partnered	Extended-family household	Bachelor’s	English only	Black	Part-time	2-5 years
**P03**	30-34	Male	Partnered	Partner/family household	Master’s	Multilingual	White	Part-time	11 + years
**P04**	35-39	Male	Not partnered	Other/unspecified	Master’s	English only	White	Part-time	6-10 years
**P05**	25-29	Male	Not partnered	Extended-family household	Bachelor’s	English only	Other racialized group	Part-time	Less than 2 years
**P06**	40-44	Male	Partnered	Partner/family household	Master’s	English only	Black	Part-time	2-5 years
**P07**	25-29	Male	Partnered	Partner/family household	Bachelor’s	English only	Black	Full-time	11 + years
**P08**	25-29	Male	Not partnered	Lives alone	Secondary school	Other/non-English	Black	Unemployed	2-5 years
**P09**	35-39	Male	Partnered	Partner/family household	Master’s	English only	Black	Full-time	6-10 years
**P10**	25-29	Male	Not partnered	Lives alone	Bachelor’s	English only	Black	Full-time	2-5 years
**P11**	30-34	Male	Not partnered	Extended-family household	Secondary school	Multilingual	Black	Part-time	Less than 2 years
**P12**	30-34	Female	Partnered	Partner/extended-family household	Bachelor’s	English only	Black	Part-time	6-10 years
**P13**	35-39	Male	Partnered	Partner/family household	Bachelor’s	Multilingual	Black	Full-time	2-5 years
**P14**	30-34	Male	Partnered	Partner/family household	Bachelor’s	English only	White	Full-time	11 + years
**P15**	25-29	Female	Not partnered	Extended-family household	College	English only	Black	Part-time	2-5 years
**P16**	30-34	Male	Partnered	Partner/family household	Bachelor’s	English only	White	Full-time	11 + years
**P17**	30-34	Female	Not partnered	Extended-family household	Bachelor’s	English only	Other racialized group	Part-time	6-10 years
**P18**	30-34	Male	Partnered	Partner household	Bachelor’s	English only	White	Full-time	11 + years
**P19**	30-34	Male	Partnered	Partner/family household	Bachelor’s	English only	White	Part-time	2-5 years
**P20**	25-29	Female	Not partnered	Lives alone	College	English only	Black	Full-time	2-5 years

### Socio-demographic characteristics of the key informants

Four key informants represent a diverse group of health and social service professionals with 1–20 years of experience, working in roles such as community support, nursing, public health and social services. They vary in age (25–43), cultural and linguistic backgrounds, and countries of birth, with immigrant clients representing 20–38% of their caseloads (Table 2).

**Table 2:** Socio-demographic characteristics of the Key Informants (n = 4).

Key informant	Age group	Gender	Job category	Practice experience	Birth region	Ethnic background	First language	Immigrant clients (%)
KI 1	25–34	Male	Community support	1–5 years	Outside Canada	Racialized	Other language	38%
KI 2	35–44	Female	Nursing / public health	11–20 years	Canada	White	English	25%
KI 3	35–44	Female	Health program / social services	11–20 years	Canada	White	English	20%
KI 4	25–34	Male	Social services	1–5 years	Outside Canada	Racialized	English	20%

### Themes

Findings indicate that healthcare access for immigrants is not a single encounter, but an ongoing, complex care journey shaped by barriers before, during, and after contact with services. Four interrelated themes characterized this journey (Table 3): Getting In, which reflected challenges initiating care due to information gaps, limited primary care attachment, transportation and distance barriers, language readiness, and fear of discrimination; Getting Through, which captured participants’ struggles to obtain timely, understandable, respectful, and culturally safe care amid language barriers, long waits, fragmented referrals, and rushed encounters; Getting On, which described the post-visit consequences of unclear follow-up, diminished trust, and constrained self-management; and Getting the System Better, which highlighted participants’ recommendations for improving immigrant healthcare access through earlier orientation, peer navigation, transportation and financial supports, targeted newcomer services, reliable language access, multilingual resources, cultural competence training, and stronger follow-up systems

**Table 3:** Themes, sub-themes and representative supporting quotations.

Themes	Sub-themes	Supporting quotations
**Getting In-Navigating the first gate to care**	**Healthcare orientation and system navigation barriers**	*“Many immigrants don’t know these services exist. There are ‘hidden rules’ that are not explained, so people arrive and simply don’t know.” (KI4)* *“I’ve been here for nine years and I still don’t have a family doctor. Honestly, I don’t even know if I will ever get one.” (KI1)*
	**Geographic and transportation barriers to accessing care**	*“Many newcomers don’t have a car or a driver’s licence, so getting to care can be difficult, especially in Northern Ontario where you may need to travel far.” (KI2)*
	**Anticipated discrimination and psychological barriers to help-seeking**	*“I didn’t seek help for my mental health… maybe I was afraid they would misunderstand me.” (P15)*
**Getting Through-Challenges during care**	**Communication difficulties and rushed healthcare encounters**	*“Ohh, the provider wants to talk fast and say everything, and I’m just like… please go bit by bit so I can understand.” (P05)*
	**Inequitable treatment and limited involvement in care**	*“It’s hard to explain things because you already feel isolated. You feel you are not treated well, not given the same services. And when communication is poor, it feels even worse.” (P02)*
	**Fragmented and financially inaccessible care**	*“In North Bay there is no HIV care provider, so people who are HIV-positive have to travel outside the city. With limited funding for virtual options, this can create out-of-pocket costs.” (KI3)* *“Sometimes the pharmacist tells me the medication is not covered. So I split the prescription or buy only part… you know, I’m always finding my way around the system because I don’t know all the rules, but I need the medicine.” (P14)*
**Getting On-Life beyond the clinic**	**Gaps in continuity, trust, and culturally safe follow-up**	*“After the appointment I didn’t hear back from anyone. I didn’t know if I should return or just wait. As a newcomer it feels like the system forgets you when you walk out.” (P18)* *“Sometimes I feel my concerns are not fully understood, and that makes it hard to trust the system.” (P06)*
	**Self-management and transnational care-seeking under constrained access**	*“With the little healthcare knowledge I had, I taught myself. I used warm water compress and took some medicine at home. I managed because I couldn’t get help quickly.” (P20)* *“It’s frustrating because my family end up spending so much money traveling back to my country for treatment, but at least we feel understood and properly cared for.” (P07)*
	**Competing responsibilities and the emotional burden of accessing care**	*“Many newcomers are balancing work, childcare, and home life without strong support. Taking time off to sit in a waiting room for 12+ hours can be impossible, which can lead to untreated conditions becoming more serious.” (KI3)*
**Getting the system Better-Strategies to advance equity and quality in care**	**Practical healthcare orientation and peer navigation**	*“Sometimes it’s hard to book things. If we had someone, like another immigrant, to help with appointments and finding resources, that would make life much easier.” (P08)*
	**Transportation and financial supports for equitable access**	*“Funders need to understand that navigating healthcare in the North takes more than a bus pass to get from point A to point B.” (KI3)* *“Some subsidy or financial plan for newcomers, especially for medications and specialist care, would really help.” (P15)*
	**Culturally safe, newcomer-focused, and language-accessible care**	*“In other cities they have community health centres that focus on immigrants. I really wish we had something like that here.” (P01)* *“We need written resources, pamphlets in simple language and in many languages about services and how to navigate the system.” (P13)*
	**Coordinated and continuous care beyond individual encounters**	*“…you know, when we come here, everything is new and fast. But care is not just one time, eh? It needs follow-up and continuity. We want to feel someone is still there, checking and helping not only today, but tomorrow too.” (P18)*

### Theme 1: Getting in-navigating the first gate to care

Getting In describes how immigrants first approached healthcare and the barriers that shaped whether they could start care at all. Entry into the system was constrained by healthcare orientation and system navigation barriers; geographic and transportation barriers to accessing care; and anticipated discrimination and psychological barriers to help-seeking.

Immigrant participants arrived without clear orientation about where to go for health services, how to register, and what was covered or not. They described healthcare rules as “hidden,” learned through trial and error rather than transparent guidance. Even when settlement supports existed, they often focused on general settlement needs rather than practical healthcare pathways (e.g., finding a family physician, understanding coverage, or navigating walk-in/ER alternatives). This uncertainty created hesitation and delays in help-seeking. One participant said: *“Many immigrants don’t know these services exist. There are ‘hidden rules’ that are not explained, so people arrive and simply don’t know.” (KI3)*

A second entry barrier was accessing primary care provider. Shortages and long waitlists left many without a family doctor, increasing reliance on episodic care. This reliance made care feel reactive rather than preventive and contributed to discontinuity. For instance, even the key informant noted: *“I’ve been here for nine years and I still don’t have a family doctor. Honestly, I don’t even know if I will ever get one.” (KI1)*

Practical access barriers, especially transportation, distance, and winter conditions, shaped whether newcomers could attend appointments and follow through on referrals. One participant explained, *“Waiting for the bus is not a joke. If you miss it, you miss it. Hmm, public transport is an issue here, and when it’s snowing the roads can be hard.” (P01)* Another participant confirmed, *“Many newcomers don’t have a car or a driver’s licence, so getting to care can be difficult, especially in Northern Ontario where you may need to travel far.” (KI2)*

Participants commonly described booking systems that felt hard to penetrate (unanswered calls, unclear instructions, and unconfirmed online bookings). Referrals sometimes meant restarting the process, repeating histories, and waiting again.

“Booking an appointment is not easy. Sometimes emails don’t go through. You call and no one answers. Hmm, the instructions are complex, and you book online but you never hear back.” (P17)

Finally, daily encounters of discrimination and mistreatment can prime newcomers to anticipate similar experiences in healthcare settings. Participants approached care worried about discrimination, describing a fear of not being welcomed or not being taken seriously. For some, positive encounters eased these concerns; for others, perceived dismissal reinforced hesitation and avoidance. *“I didn’t seek help for my mental health… maybe I was afraid they would misunderstand me.” (P15)*

While these barriers shaped whether immigrants could get into the healthcare system, participants’ experiences also showed that reaching care did not necessarily mean that access challenges were resolved. The next theme therefore moves from the challenges of entering care to the barriers immigrants encountered during care, where access depended not only on reaching services but also on how care was delivered, explained, and experienced.

### Theme 2: Getting through-challenges during care

Getting Through captures what happened after participants entered the system: the struggle to secure timely care, understand instructions, and feel respected and safe. Experiences were dominated communication difficulties and rushed healthcare encounters; inequitable treatment and limited involvement in care; and fragmented and financially inaccessible care. Inside the encounter, participants highlighted communication gaps. Language barriers, fast speech, and complex medical terms left some unsure about diagnosis, medications, or self-care instructions. Language readiness influenced entry by shaping whether participants could confidently communicate needs and understand next steps. Even when individuals spoke English, accents and medical terminology created misunderstandings and extra effort during first contact with services. One participant noted, *“Ohh, the provider wants to talk fast and say everything, and I’m just like… please go bit by bit so I can understand.” (P05)* A KI added, *“Even if someone speaks some English, medical terminology can be much harder. Interpretation helps, but some terms don’t translate well, so it takes longer to explain.” (KI2)*

Long waits, particularly for mental health supports and specialist services, created exhaustion and discouragement. Even after waiting, participants often described appointments that felt rushed, with instructions delivered quickly and without checking understanding. A participant said, *“I waited for hours, and when I finally saw the doctor it felt rushed. I didn’t fully understand what to do after.” (P18)* Another participant added, *“In walk-in clinics they rush you. Sometimes it’s only two to five minutes.” (P06)*

Several participants described feeling de-prioritized, overlooked, or not fully involved in decisions. Some responded by becoming passive in care, accepting decisions without full understanding because they wanted relief, feared conflict, or did not know how to advocate within unfamiliar structures. Key informants also pointed to systemic limitations in diversity, equity, and inclusion that can shape patients’ experiences of respect and safety. For instance, one participant explained, *“It’s hard to explain things because you already feel isolated. You feel you are not treated well, not given the same services.” (P02) A KI explained, “Sometimes stereotypes show up in the system. I have seen situations where someone didn’t want to serve a certain group of people.” (KI1)*

Care coordination was a persistent issue. Some participants described moving between multiple sites, repeating steps, and traveling out of town for specialist care, such as cardiology and dermatology. When systems were fragmented, the work of “holding care together” shifted to newcomers with limited time, resources, transportation, and support. One healthcare provider said, *“In North Bay there is no HIV care provider, so people who are HIV-positive have to travel outside the city. With limited funding for virtual options, this can create out-of-pocket costs.” (KI3) Another participant added “… I had to travel to access specialized care, including cardiology, dermatology, and other specialist services.” (P13)*

Affordability pressures emerged most clearly at the point of medication access, where coverage confusion and out-of-pocket costs led to “workarounds” such as splitting prescriptions or purchasing partial supplies. *“Sometimes the pharmacist tells me the medication is not covered. So I split the prescription or buy only part… you know, I’m always finding my way around the system because I don’t know all the rules, but I need the medicine.” (P14)*

While Getting Through shows that entering the healthcare setting did not guarantee timely, clear, or culturally safe care, participants’ accounts also revealed that challenges continued after the appointment ended. The effects of rushed explanations, fragmented referrals, medication costs, and unclear instructions often followed participants back into their everyday lives, where they had to interpret next steps, manage prescriptions, arrange follow-up, and continue navigating the system with limited support. The next theme therefore shifts attention to what happened after the clinic, examining how immigrants carried the responsibility of continuing care beyond the healthcare encounter.

### Theme 3: Getting on-life beyond the clinic

Getting On reflects the experiences of care after the visit, particularly gaps in continuity, trust, and culturally safe follow-up; self-management and transnational care-seeking under constrained access; and competing responsibilities and the emotional burden of accessing care. Many participants described a “handoff gap”: once the appointment ended, they were unsure what came next and who was responsible for ongoing care. Without proactive follow-up, individuals felt alone in managing symptoms, monitoring recovery, and deciding when to return. For example, one participant noted, *“After the appointment I didn’t hear back from anyone. I didn’t know if I should return or just wait. As a newcomer it feels like the system forgets you when you walk out.” (P18)*

These experiences influenced trust. For some, healthcare became a last resort used only for severe symptoms, reinforcing emergency department reliance and delaying earlier intervention. Key informants noted that lack of language access and limited cultural recognition can make care feel unsafe and isolating, which further discourages use. A participant described*, “Sometimes I feel my concerns are not fully understood, and that makes it hard to trust the system.” (P06)* A KI added, *“Many newcomers cannot get care in their own language and may not have access to interpreters. When cultural norms and non-Western beliefs are not respected alongside Western medicine, care can feel isolating and unsafe.” (KI3)*

In response, participants described self-care and self-management strategies such as resting, exercise, seeking online information, relying on community encouragement, and, at times, self-medicating while waiting for appointments. While these strategies reflected resilience, they also highlighted unmet needs and structural barriers. For instance, a study participant explained, *“With the little healthcare knowledge I had, I taught myself. I used warm water compress and took some medicine at home. I managed because I couldn’t get help quickly.” (P20)* Four participants reported choosing to return to their home countries in search of what they perceive to be higher-quality care. Participants noted that this decision often places an additional financial burden on them and their families. Participants noted, *“It’s frustrating because my family end up spending so much money traveling back to my country for treatment, but at least we feel understood and properly cared for.” (P07)*

Finally, the emotional aftermath, frustration, loneliness, and stress were frequently linked to long waits, repeated barriers, and limited social supports. Key informants emphasized that work demands, childcare, and lack of networks often made lengthy waiting-room care impossible, increasing the risk of untreated or worsening conditions. For example, one KI explained, *“Many newcomers are balancing work, childcare, and home life without strong support. Taking time off to sit in a waiting room for 12+ hours can be impossible, which can lead to untreated conditions becoming more serious.” (KI3)*

### Theme 4: Getting the system better-strategies to advance equity and quality in care

Getting the system better summarizes suggested strategies to improve healthcare services for immigrants. Recommendations focused practical healthcare orientation and peer navigation; transportation and financial supports for equitable access; culturally safe, newcomer-focused, and language-accessible care; coordinated and continuous care beyond individual encounters.

Participants and key informants emphasized that orientation should be practical and honest, explaining not only where to go but what to expect (including wait times and eligibility). *“We have to be transparent and accountable when we share information… we need to tell people there are barriers to services.” (KI1)*

Peer navigators, particularly other immigrants, were viewed as a realistic way to reduce confusion and increase confidence in booking and referrals. For instance, a participant suggested, *“Sometimes it’s hard to book things. If we had someone, like another immigrant, to help with appointments and finding resources, that would make life much easier.” (P08)*

Transportation was described as a structural equity issue in northern contexts. Improving routes, proximity of stops, and broader travel supports could directly increase timely access. For example, one participant explained, *“… It would really help if the bus came closer to our area. Right now, we walk about 15 minutes to the nearest stop. That is hard with appointments or bad weather.” (P18)* Another KI added, *“Funders need to understand that navigating healthcare in the North takes more than a bus pass to get from point A to point B.” (KI3)*

Some participants also advocated for better cost protection for medications and specialist services. For instance one participant’s quote highlights, *“Some subsidy or financial plan for newcomers, especially for medications and specialist care, would really help.” (P15)*

Culturally safe care was described as both relational (being treated with respect, seen, and believed) and structural (having newcomer-focused services, telemedicine, mental health supports, cultural competence training and diverse teams). One participant noted, *“In other cities they have community health centres that focus on immigrants. I really wish we had something like that here.” (P01)* A KI added, *“Across healthcare there is a real need for cultural competence. Some clients fear stigma and worry about how they will be treated, especially in the emergency department.” (KI2)*

Participants strongly emphasized the need for interpreters and multilingual written materials that explain coverage, steps, and local pathways, especially in emergency care settings. One participant suggested, *“Yeah, having a translator for common languages would help… and maybe a translating app for less common languages too.” (P11) A KI* reinforced this*, “Language services and interpreters that community organizations can access at low or no cost are important for culturally safe care.” (KI3)*

Again, the participants repeatedly called for practical written materials, simple, multilingual pamphlets and printed information that explain services, coverage, and steps for care. Key informants noted that even when interpreters exist, printed resources are often missing. *“We need written resources, pamphlets in simple language and in many languages about services and how to navigate the system.” (P13)*

Finally, they highlighted continuity: follow-up and coordination were seen as essential to prevent people from feeling “dropped” after a single visit*. “… you know, when we come here, everything is new and fast. But care is not just one time, eh? It needs follow-up and continuity. We want to feel someone is still there, checking and helping not only today, but tomorrow too.” (P18)*

Together, these themes highlight the challenges newcomers face across the continuity of care. Participants’ narratives revealed that barriers were cumulative and interconnected, beginning with uncertainty and limited access to primary care, which often led individuals to rely on walk-in clinics and emergency departments. Once in the system, rushed encounters and inadequate cultural safety eroded trust, while unclear follow-up processes shifted the responsibility for care coordination onto newcomers and their families.

## Discussion

This study highlights that immigrants’ experiences with healthcare in rural and northern Ontario are shaped by a complex and ongoing care journey rather than a single point of access. This study shows that critical to rural migration are concerns over access to care which attributed to challenges in transportation for immigrants who cannot drive, low language abilities for those that English is not the first language, fear of being discriminated against or not believed at the point of care, and the lack role clarity within the healthcare system. These issues are foundational to concerns over equity and diversity given that most immigrants are from diverse socio-cultural background. In addition, concerns over lack of culturally sensitive and an ecosystem for cultural respect are barriers to accessing healthcare for immigrants to rural communities.

### Information and communication is critical to overcoming barriers and navigating healthcare system

In the present study, information gaps among immigrants, especially regarding how to enroll with a family physician and navigate provincial referral pathways, mirror persistent inequities documented in Canadian research [17,18]. A recent national survey found that having a regular provider is uneven across racialized groups, underscoring structural barriers to entry and continuity [18]. This finding confirms the experiences shared by participants in our current study. Thus, in 2023, adults identifying as Arab, Black, Korean, Latin American, and Chinese were less likely than non-racialized adults to have a regular healthcare provider, thereby compounding newcomers’ early access challenges [18]. Evidence highlights that system literacy and fragmented information ecosystems intersect with migration-related stressors, amplifying unmet needs despite formal coverage [19,20]. These findings position information readiness not as an individual deficit but as a system-level accessibility issue requiring plain‑language enrollment supports, culturally responsive navigation, and proactive outreach in settlement contexts [18,20].

Our study found that language readiness impacts communications and intensifies fear of discrimination, preventing many immigrants from accessing healthcare. Moreover, immigration itself organizes disadvantage through policy and institutional norms, and healthcare organizations must actively counter language exclusion and bias through interpretation, culturally safe communication, and anti-racism commitments embedded in care delivery [21,22]. Furthermore, studies in Canada have identified language gaps as critical barriers to healthcare access, noting that newcomers’ health information needs exceed available linguistically appropriate resources, which drives avoidance, misunderstandings, and delayed care [23]. Similarly, findings of a review study converge on discrimination and cultural incongruence as recurring barriers, where concerns about stigma, differential treatment, or administrative gatekeeping deter engagement even when services are nominally available [19,20]. Addressing these barriers requires institutional accountability, routine collection of disaggregated data, funded interpreter services, and targeted quality improvement that treats language and anti-discrimination as non-negotiable dimensions of safety [20,22].

Once entered in the healthcare systems, our study revealed that communication gaps during clinical encounters often shape newcomers’ healthcare experiences and compound existing access challenges. These findings highlight how communication barriers operate beyond simple measures of English proficiency and instead reflect structural and relational dynamics within healthcare encounters. Many immigrants often describe difficulties related to language barriers, particularly difficulties understanding complex medical terminology, which can leave them uncertain about diagnoses, medication instructions, recommended treatment, and other care instructions [17,24]. Even among immigrants who speak English, the accents and fast speech, as well as the inability to explain health problems using clinical language, not only influence the quality of interactions with healthcare workers but also potentially lead to misunderstandings that may compromise the quality of care given, as well as impact patient safety, satisfaction and health outcomes [17,25,26]. As more immigrants settle in these rural communities, addressing communication barriers through plain-language communication, increasing the availability of professional interpretation services, and raising healthcare providers’ awareness of linguistic diversity are critical to improving newcomer healthcare experiences in these communities.

### Healthcare shortages and lack of coordination

Participants reported how long wait times, staff shortages, and other systemic barriers affected their access to healthcare in Canada. Long waits and staff shortages reflect broader capacity strains in Canadian primary and community care, with downstream consequences for newcomers who often lack established networks to buffer delays. Research emphasizes ongoing recruitment challenges for nurses, residential care, and home health roles, which intensifies bottlenecks, including long wait times, in access points where newcomers seek first-line services [17]. These pressures are magnified outside urban cores, where transportation and distance impose structural constraints on rural and northern access, compounding appointment scarcity with geographic isolation and limited transit options, consistent with national analyses of unmet needs and provider shortages [18]. Evidence syntheses similarly note that service availability intersects with place, where rural contexts heighten logistical burdens and foregone care, especially for newly arrived populations with limited car ownership and flexible employment arrangements [20]. A capacity response must integrate workforce stabilization with transportation supports and mobile or virtual care models tailored to rural settlement patterns [18,20].

Another key finding was participants’ experiences of being overlooked or insufficiently involved in healthcare decision-making. They described accepting care decisions without full understanding, motivated by a desire for symptom relief, fear of conflict, or uncertainty about how to express their needs and advocate for themselves within unfamiliar healthcare structures. These responses reflect adaptive strategies within power-imbalanced encounters rather than a lack of interest or engagement. These findings have been corroborated by previous studies where immigrants often felt excluded from the health decision-making process, with healthcare workers deciding on the plan of care for the client [24,27]. This lack of involvement is usually attributed to communication and language barriers that exist between healthcare providers and immigrants [17,27]. In rural settings, where provider choice may be limited, participants may feel particularly constrained in expressing concerns or seeking clarification, further reducing opportunities for shared decision-making [24,26]. In these settings, where access to interpretation services and culturally responsive care may be limited, communication barriers and exclusion from health decisions may foster fear, anxiety and mistrust of the healthcare system, potentially contributing to the underutilization of healthcare services [24,27,28].

Care coordination also emerged as a central challenge for participants. Newcomers frequently described moving between multiple care sites and having to travel out of town to access specialist services. These findings align with other studies, documenting challenges with continuity and coordination of care among immigrant populations and highlight how living in rural communities compounds these issues [24,29]. For newcomers unfamiliar with how the Canadian healthcare system is organized, navigating fragmented care pathways requires substantial informational support and frequently results in reliance on one’s social networks [24,27]. In addition, many immigrants often describe a lack of awareness about insurance coverage and co-payments, especially for prescribed medications. These, in addition to the lack of consistency in insurance coverage, lead to misunderstandings and confusion about medication access in the country [30]. These challenges highlight the need for system-level interventions that improve health system navigation.

In this study, participants reported leaving clinic visits with a limited understanding of who was responsible for monitoring their progress, when to return, or what to do if symptoms persisted. These narratives highlight a broader “handoff gap” in care transitions. Once the appointment ended at the clinic, participants faced uncertainty, a lack of follow-up, and unclear responsibility for ongoing care. This ambiguity is not merely an informational problem; it signals a lack of relational continuity and accountability within the health care system. This aligns with recent studies of migrants and refugees in high-income countries, which show that poorly defined follow-up and fragmented care pathways undermine continuity of care, particularly for migrant populations facing additional structural and communication barriers [31–33]. Participants’ tendency to reserve hospital care as a last resort, often limited to severe symptoms and emergency department visits, reflects how episodic, poorly coordinated care can unintentionally push people toward crisis-driven service use rather than preventive or early intervention.

### Cultural safety and system improvements

Across participants’ accounts, trust appears as a central concern shaping how care is perceived and navigated. They expressed that their concerns were not fully understood, particularly when language and cultural norms were not acknowledged or accommodated. Key informants observed that limited language access and lack of respect for non-Western beliefs can render care “unsafe”. This resonates with recent scholarship on cultural safety, racism, and structural inequities in healthcare [31]. When individuals repeatedly experience not being heard, not being believed, or not having their worldviews reflected in care plans, trust erodes, not only with individual providers but with the system as a whole. This erosion helps explain why some participants framed the hospital as a “last option,” reinforcing patterns of late presentation and acute care dependence that continue to be documented among newcomers in high-income settings. Canadian qualitative studies document how immigrants’ experiences of dismissed concerns, poor communication, and culturally insensitive care contribute to frustration, disengagement, and diminished confidence in health services [34,35]. Syntheses of Canadian evidence further show that these relational and communicative failures accumulate over time, undermining trust and continuity of care among newcomers [24].

In response to these systemic gaps, participants turned to self-care and self-management strategies, including online information seeking, peer and community support, self-medication and returning to their home countries. These practices can be understood as both expressions of agency and symptoms of constrained choice. Studies show that immigrants frequently rely on informal and digital sources of health information when formal services are difficult to access or perceived as unresponsive [24,35]. While these strategies may support autonomy and continuity in the absence of timely care, evidence also suggests that limited health literacy and lack of trustworthy guidance can lead to confusion, anxiety, or potentially unsafe treatment decisions [24,36]. The emotional labour of “getting on” is also evident in participants’ descriptions of feeling alone with their symptoms, uncertain about what was “normal,” and reluctant to “bother” providers or re-expose themselves to challenging encounters. This echoes emerging evidence that repeated experiences of discrimination, dismissal, or language-based exclusion in healthcare contribute to anticipatory stress and avoidance among racialized and migrant communities [35]. For newcomers with limited social networks, this emotional toll is intensified by isolation and the lack of informal supports who can help interpret symptoms, navigate services, or advocate within the system [37].

Participants described multiple, practical strategies to improve the health-care system for immigrants living in Northern communities. They emphasized the need for earlier and clearer orientation to local services, supported by peer navigation to help newcomers understand where to go and how to access care. Participants’ suggestions align with evidence showing that immigrants frequently face communication, cultural, and system‑navigation barriers, underscoring the need for community‑based programming and culturally competent care to improve access [20]. Thus, to make care more welcoming and effective, they called for culturally safe, targeted newcomer services, improved language access (including interpretation), and multilingual written resources. They also recommended strengthening provider cultural competence through training and improving continuity through more reliable follow-up systems, so immigrants are not left to manage complex health needs on their own after an initial visit. Finally, participants also highlighted structural barriers, especially transportation and cost, and recommended transportation solutions alongside stronger financial protections to reduce out-of-pocket burdens. Several studies have identified improvements in public transportation as a key strategy for enhancing access to healthcare services, particularly for newcomers who often do not own a car during their early settlement period [20,21].

### Policy implications

The study implications are that governments should treat immigrant healthcare access as a shared systems-level equity responsibility rather than an issue left to individuals to navigate alone. At the federal level, immigration, settlement, and health-transfer policies should support practical health-system orientation, consistent language-access standards, and dedicated funding for newcomer navigation, interpretation, medication affordability, and northern/rural access supports. Regional and provincial governments should translate these commitments into service design by strengthening primary care attachment, expanding community-based newcomer health models, requiring culturally safe practice across care settings, improving referral and follow-up coordination, and aligning mental health, specialist, virtual-care, and transportation supports so that access does not depend on personal resources, English proficiency, or prior system knowledge. At the municipal level, implications include embedding health navigation within settlement and community services, improving transit and community transport links to clinics and hospitals, distributing multilingual information through libraries, schools, community organizations, and faith/community hubs, and building local partnerships that make care pathways visible, trusted, and easier to use. Overall, the findings point to the need for shared accountability across jurisdictions, with federal policy and funding, regional/provincial service planning, and municipal implementation working together to move immigrant healthcare from a reactive, fragmented process toward a coordinated, equitable, and culturally safe continuum of care.

### Strengths and limitations

A key strength of this study is that its qualitative descriptive design was well suited to exploring immigrants’ healthcare navigation experiences in depth and in participants’ own words. The inclusion of both immigrant participants and KIs strengthened the study by capturing perspectives from service users and those working with immigrants in northern healthcare contexts. However, the study also has some limitations. Because the study was conducted in one northern Ontario community, the findings may not fully reflect immigrant experiences in other rural, northern, urban, or larger resettlement contexts. The small purposive sample limits broad transferability, although this is appropriate for qualitative inquiry. Eligibility was restricted to participants who could speak English or French and who had already accessed healthcare services in North Bay, which may have excluded immigrants with greater language barriers, those who were more socially isolated, or those unable to access care at all. Although immigrant participants were offered the option of participating either in person at a location of their choice or virtually, all elected to complete their interviews online. Participants may have preferred virtual interviews because of convenience, work and family responsibilities, transportation challenges, privacy concerns, or greater comfort speaking from their own environment. While the online format may have enhanced participation by reducing travel and scheduling barriers, it may also have limited the researchers’ ability to observe non-verbal cues and build rapport to the same extent as in-person interviews, potentially affecting the depth and richness of some responses. Finally, the study relied on self-reported experiences, which may be influenced by recall, interpretation, or willingness to disclose sensitive experiences such as discrimination, fear, or dissatisfaction with care.

## Conclusion

This study shows that immigrant healthcare access is best understood as a continuous and interconnected care journey rather than a single point of service use. Therefore, improving healthcare access for immigrants in rural and Northern communities requires coordinated, immigrant-responsive strategies, including early health system orientation, peer navigation, reliable language supports, multilingual resources, transportation and financial assistance, culturally safe newcomer-focused services, provider cultural competence training, and stronger follow-up processes. Together, these measures can support a more accessible, equitable, and responsive healthcare system for immigrant communities.

## Supporting information

S1 Appendix A Interview Guide-Immigrants. (DOCX)

S2 Appendix B Interview Guide-Key Informants. (DOCX)

## References

[1] Statistics Canada. Immigrants make up the largest share of the population in over 150 years and continue to shape who we are as Canadians. Stat. Canada. 2022 [cited 2025 Feb 4]. Available from: https://www150.statcan.gc.ca/n1/daily-quotidien/221026/dq221026a-eng.htm

[2] Ministry of Finance. Ontario’s Long-term Report on the Economy. Minist. Financ. Toronto, ON; 2020. Available from: https://files.ontario.ca/mof-long-term-report-book-2020-06-03-en.pdf

[3] Immigration Refugees and Citizenship Canada. 2023 Settlement Outcomes Report. IRCC. 2025 [cited 2026 Jan 9]. Available from: https://www.canada.ca/en/immigration-refugees-citizenship/corporate/settlement-resettlement-service-provider-information/2023-settlement-outcomes-report.html

[4] Statistics Canada. Focus on Geography Series, 2021 Census of Population: North Bay, City. Stat. Canada. 2025 [cited 2026 Jan 9]. Available from: https://www12.statcan.gc.ca/census-recensement/2021/as-sa/fogs-spg/Page.cfm?lang=e&topic=9&dguid=2021A00053548044

[5] Haugen S, Mcnally R, Hallström LK. Settling beyond big cities: A scoping review of the Canadian literature on immigration to rural and smaller communities. Can Geogr. 2024;68:456–67.

[6] Mabee K. Perceptions of Community Welcoming: Measuring Individuals ’ Experiences and Satisfaction with Services in North Bay: What Makes a Welcoming Community? (Series). 2022. Available from: https://www.northernpolicy.ca/upload/documents/publications/briefing-notes/mm2022/en-individualsurvey-northbay-2022-12-08-.pdf

[7] Robert A-M, Gilkinson T. Mental health and well-being of recent immigrants in Canada: Evidence from the Longitudinal Survey of Immigrants to Canada (LSIC). Governement of Canada. 2012 [cited 2021 Sep 21]. Available from: https://www.canada.ca/en/immigration-refugees-citizenship/corporate/reports-statistics/research/mental-health-well-being-recent-immigrants-canada-evidence-longitudinal-survey-immigrants-canada-lsic.html

[8] Haugen S, McNally R, Hallström LK. Settling beyond big cities: A scoping review of the Canadian literature on immigration to rural and smaller communities. Can Geogr. 2024;:1–12.

[9] Creswell J. Research design: Qualitative, quantitative and mixed methods approaches. SAGE Publications; 2014.

[10] Guba EG, Lincoln YS. Competing paradigms in qualitative research. Handbook of qualitative research. 1994. p. 105–17.

[11] North Bay Ontario Cabada. About North Bay. [cited 2026 May 21]. Available from: https://northbay.ca/our-community/about-north-bay/

[12] City of North Bay. Health Care. City North Bay. 2026. Available from: https://northbayimmigration.ca/living-in-north-bay/health-care/

[13] Braun V, Clarke V. Toward good practice in thematic analysis: Avoiding common problems and be(com)ing a knowing researcher. Int J Transgend Health. 2022;24(1):1–6. doi: 10.1080/26895269.2022.2129597 36713144 PMC9879167

[14] Braun V, Clarke V. Reflecting on reflexive thematic analysis. Qualitative Research in Sport, Exercise and Health. 2019;11(4):589–97. doi: 10.1080/2159676x.2019.1628806

[15] Braun V, Clarke V. Using thematic analysis in psychology. Qual Res Psychol. 2006;3:77–101.

[16] Lincoln YS, Guba EG. But is it rigorous? Trustworthiness and authenticity in naturalistic evaluation. New Dir Eval. 1986;1986:73–84.

[17] Pandey M, Kamrul R, Michaels CR, McCarron M. Identifying barriers to healthcare access for new immigrants: A qualitative study in Regina, Saskatchewan, Canada. J Immigr Minor Health. 2022;24(1):188–98. doi: 10.1007/s10903-021-01262-z 34426892 PMC8382209

[18] Statistics Canada. Health of Canadians: Access to health care. Stat. Canada. 2025 [cited 2026 May 21]. Available from: https://www150.statcan.gc.ca/n1/pub/82-570-x/2024001/section4-eng.htm

[19] Sundareswaran M, Martignetti L, Purkey E. Barriers to primary care among immigrants and refugees in Peterborough, Ontario: A qualitative study of provider perspectives. BMC Prim Care. 2024;25(1):199. doi: 10.1186/s12875-024-02453-x 38840096 PMC11151623

[20] Tsai P-L, Ghahari S. Immigrants’ experience of health care access in Canada: A recent scoping review. J Immigr Minor Health. 2023;25(3):712–27. doi: 10.1007/s10903-023-01461-w 36870008

[21] Lane G, Vatanparast H. Adjusting the Canadian healthcare system to meet newcomer needs. Int J Environ Res Public Health. 2022;19(7):3752. doi: 10.3390/ijerph19073752 35409441 PMC8997438

[22] Wiedmeyer M-L, Machado S, Tayyar E, Sierra-Heredia C, Bozorgi Y, Hagos S, et al. How immigration shapes health disadvantages and what healthcare organizations can do to deliver more equitable care. Healthc Manage Forum. 2025;38(1):16–22. doi: 10.1177/08404704241265675 39045719 PMC11650901

[23] Mianji F, Tomaro J, Kirmayer LJ. Linguistic and cultural barriers to access and utilization of mental health care for Farsi-speaking newcomers in Quebec. IJMHSC. 2020;16(4):495–510. doi: 10.1108/ijmhsc-08-2019-0074

[24] Bajgain BB, Bajgain KT, Badal S, Aghajafari F, Jackson J, Santana M-J. Patient-Reported Experiences in Accessing Primary Healthcare among Immigrant Population in Canada: A Rapid Literature Review. Int J Environ Res Public Health. 2020;17(23):8724. doi: 10.3390/ijerph17238724 33255379 PMC7727859

[25] Gu Y, Shah AP. A systematic review of interventions to address accent-related communication problems in healthcare. Ochsner J. 2019;19(4):378–96. doi: 10.31486/toj.19.0028 31903062 PMC6928667

[26] Wang L, Kwak M-J. Immigration, barriers to healthcare and transnational ties: A case study of South Korean immigrants in Toronto, Canada. Soc Sci Med. 2015;133:340–8. doi: 10.1016/j.socscimed.2014.11.039 25481040

[27] Floyd A, Sakellariou D. Healthcare access for refugee women with limited literacy: Layers of disadvantage. Int J Equity Health. 2017;16(1):195. doi: 10.1186/s12939-017-0694-8 29126420 PMC5681803

[28] Brandenburg L, Gabow P, Steele G, Toussaint J, Tyson BJ, Brandenburg L. Innovation and Best Practices in Health Care Scheduling. 2015.

[29] Lum ID, Swartz RH, Kwan MYW. Accessibility and use of primary healthcare for immigrants living in the Niagara Region. Soc Sci Med. 2016;156:73–9. doi: 10.1016/j.socscimed.2016.03.024 27017093

[30] Antonipillai V, Schwartz L, Wahoush O, Baumann A. Patient and provider perspectives on how migrants access prescription drugs in Ontario: Implications for health policy and practice. SSM - Qualitative Research in Health. 2022;2:100063. doi: 10.1016/j.ssmqr.2022.100063

[31] Curtis E, Jones R, Tipene-Leach D, Walker C, Loring B, Paine S-J, et al. Why cultural safety rather than cultural competency is required to achieve health equity: A literature review and recommended definition. Int J Equity Health. 2019;18(1):174. doi: 10.1186/s12939-019-1082-3 31727076 PMC6857221

[32] Filler T, Jameel B, Gagliardi AR. Barriers and facilitators of patient centered care for immigrant and refugee women: A scoping review. BMC Public Health. 2020;20(1):1013. doi: 10.1186/s12889-020-09159-6 32590963 PMC7318468

[33] Lebano A, Hamed S, Bradby H, Gil-Salmerón A, Durá-Ferrandis E, Garcés-Ferrer J, et al. Migrants’ and refugees’ health status and healthcare in Europe: A scoping literature review. BMC Public Health. 2020;20(1):1039. doi: 10.1186/s12889-020-08749-8 32605605 PMC7329528

[34] Dastjerdi M, Olson K, Ogilvie L. A study of Iranian immigrants’ experiences of accessing Canadian health care services: A grounded theory. Int J Equity Health. 2012;11:55. doi: 10.1186/1475-9276-11-55 23021015 PMC3519565

[35] Woodgate RL, Busolo DS, Crockett M, Dean RA, Amaladas MR, Plourde PJ. A qualitative study on African immigrant and refugee families’ experiences of accessing primary health care services in Manitoba, Canada: it’s not easy!. Int J Equity Health. 2017;16(1):5. doi: 10.1186/s12939-016-0510-x 28068998 PMC5223444

[36] Tørris C, Nortvedt L. Health literacy and self-care among adult immigrants with type 2 diabetes: A scoping review. BMC Public Health. 2024;24(1):3248. doi: 10.1186/s12889-024-20749-6 39578821 PMC11583541

[37] Anjorin O, Agyapong-Opoku G, Sodunke T, Harri BI, Duke AEE, Ale OAJ, et al. Immigrants’ experiences of barriers and enablers to mental health services in Canada - A scoping review. J Immigr Minor Health. 2026;:10.1007/s10903-026-01868–1. doi: 10.1007/s10903-026-01868-1 41652179

