# Supplementary material for: Navigating healthcare access and utilization in rural and Northern Ontario: Immigrants’ experiences amid regionalization and dispersal policies
Source: PLoS One. 2026 Nov 11;21(11):e0362085. doi: 10.1371/journal.pone.0362085 (PMC13754121; doi:10.1371/journal.pone.0362085)
Supplement: Supplementary file 1 [file pone.0362085.s001.docx]

**Appendix A: Interview Guide-Immigrants**

**Introduction**

Thank you for agreeing to participate in this interview. We are conducting this study to better understand the experiences of immigrants accessing and using healthcare services in Northern Ontario, particularly in the North Bay region. Your perspectives and experiences are valuable and will help us identify factors that support or hinder access to healthcare, as well as opportunities to improve services for immigrant populations. Before we begin, I would like to remind you that your participation is entirely voluntary. You may choose not to answer any question and may stop the interview at any time without consequence. With your permission, this interview will be audio-recorded to ensure that your responses are accurately captured. All information you provide will be kept confidential, and any identifying information will be removed from reports and publications arising from this research. The interview is expected to take approximately 45 to 60 minutes. There are no right or wrong answers; we are interested in your personal experiences and opinions. Please feel free to share as much detail as you are comfortable providing. If you have any questions before we begin, I would be happy to answer them. Do you have any questions before we start, and do I have your permission to begin the interview?

1. Can you describe some of the health or healthcare challenges you have faced since moving to North Bay?
2. What barriers have you encountered when trying to access healthcare services in this region, and how have they impacted your well-being?
3. Can you tell me about any difficulties experienced related to language, cultural differences, or healthcare system navigation? How did you manage them?
4. What types of healthcare services or support do you believe are missing or inadequate for newcomers in North Bay?
5. Can you share any positive experiences or strategies that have helped you access healthcare more effectively in this region?
6. What recommendations do you have for improving healthcare accessibility and quality for immigrants in North Bay?
7. How do you think community organizations, policymakers, and healthcare providers can better support the health and well-being of newcomers in these areas?
8. What are your top priorities when it comes to developing healthcare strategies/interventions that effectively support immigrants in North Bay?

**About You:**

1. Your DOB……… (X years)
2. Your gender …….
3. Marital status:....
4. You family (family size: spouse/children, parents etc.) ………
5. Your highest level of education you completed……….
6. Language you mainly speak at home………….
7. People living in Canada come from many different cultural and racial backgrounds. Are you? (E.g., East Asian, West Asian, Black, Latin American) …………
8. Your Employment status (E.g., FT, PT, at Study, Retired, unemployed) ……….
9. Length of stay in Canada since immigration--------------year(s)
10. Health insurance

Lastly, thank you so much for your time and sharing your experiences in this important topic. I greatly appreciate your willingness to talk with me today.
