# Supplementary material for: Navigating healthcare access and utilization in rural and Northern Ontario: Immigrants’ experiences amid regionalization and dispersal policies
Source: PLoS One. 2026 Nov 11;21(11):e0362085. doi: 10.1371/journal.pone.0362085 (PMC13754121; doi:10.1371/journal.pone.0362085)
Supplement: Supplementary file 2 [file pone.0362085.s002.docx]

**Appendix B: Interview Guide-Key Informants**

**Introduction**

Thank you for agreeing to participate in this interview. We are conducting this study to better understand the experiences of immigrants accessing and using healthcare services in Northern Ontario, particularly in the North Bay region. Your perspectives and experiences are valuable and will help us identify factors that support or hinder access to healthcare, as well as opportunities to improve services for immigrant populations. Before we begin, I would like to remind you that your participation is entirely voluntary. You may choose not to answer any question and may stop the interview at any time without consequence. With your permission, this interview will be audio-recorded to ensure that your responses are accurately captured. All information you provide will be kept confidential, and any identifying information will be removed from reports and publications arising from this research. The interview is expected to take approximately 45 to 60 minutes. There are no right or wrong answers; we are interested in your personal experiences and opinions. Please feel free to share as much detail as you are comfortable providing. If you have any questions before we begin, I would be happy to answer them. Do you have any questions before we start, and do I have your permission to begin the interview?

1. **What are the most common health and healthcare challenges that newcomers face in North Bay?**
2. **From your experience, what barriers prevent newcomers from accessing healthcare services in this community?**
3. **How do language and cultural differences impact the ability of newcomers to navigate the healthcare system?**
4. **What existing programs or initiatives have been effective in supporting newcomers’ health and well-being?**
5. **What role do settlement services play in connecting newcomers with healthcare resources, and what gaps still exist?**
6. **What policy or system-level changes would improve healthcare access and outcomes for newcomers in North Bay?**
7. **How can healthcare providers, community organizations, and policymakers collaborate more effectively to address newcomers’ health needs?**
8. What are the **top-priority interventions**/strategies that can be developed to better support immigrants in North Bay?

**Socio-demographic data**

1. Age:	years
2. Gender:
3. Job title:
4. Years of practice: ..........years
5. Country of birth:
6. Ethnic background:
7. First language:
8. Approximate proportion of immigrants in your practice:

Lastly, thank you so much for your time and for sharing your experiences in this important topic. I greatly appreciate your willingness to talk with me today.
